# Reproductive outcomes and risk factors of women with septate uterus after hysteroscopic metroplasty

**DOI:** 10.3389/fendo.2023.1063774

**Published:** 2023-06-08

**Authors:** Yanan Chang, Minghong Shen, Sha Wang, Zhengchen Guo, Hua Duan

**Affiliations:** ^1^ Department of Minimally Invasive Gynecology, Beijing Obstetrics and Gynecology Hospital, Capital Medical University. Beijing Maternal and Child Health Care Hospital, Beijing, China; ^2^ Department of Gynecology, Shengli Clinical Medical College of Fujian Medical University, Fujian Provincial Hospital, Fuzhou, Fujian, China

**Keywords:** septate uterus, hysteroscopic metroplasty, reproductive outcomes, live birth, clinical pregnancy

## Abstract

**Background:**

Hysteroscopic metroplasty of the uterine septum has been the standard treatment strategy to improve reproductive outcomes, but there are still controversies about the appropriateness of metroplasty. In addition, there have been few studies of the factors related to reproductive outcomes of women after surgery. The study aimed to evaluate the reproductive outcomes and the associated risk factors that influence reproductive outcomes after hysteroscopic metroplasty of women with septate uterus and the desire to conceive.

**Methods:**

This study was an observational study. Cases were screened by searching electronic patient files, and demographic factors were collected. We conducted telephone follow-ups to collect the postoperative reproductive outcomes. The primary outcome of this study was live birth, and secondary outcomes were ongoing pregnancy, clinical pregnancy, early miscarriage, and preterm birth. Demographic variables included patients’ age, body mass index (BMI), the type of septum, infertility and miscarriage history, and complications including intrauterine adhesions, endometrial polyps, endometriosis, and adenomyosis were collected to perform univariate and multivariate analyses to predict the risk factors of reproductive outcomes after surgery treatment.

**Results:**

In total, 348 women were evaluated and followed up. There were 95 cases (27.3%, 95/348) with combined infertility, 195 cases (56.0%, 195/348) with miscarriage history, and cases combined with intrauterine adhesions, endometrial polyps, endometriosis, and adenomyosis were 107 (30.7%, 107/348), 53 (15.2%, 53/348), 28 (8.0%, 28/348), and 5 (1.4%), respectively. Following surgery, the live birth rate and clinical pregnancy rate were significantly higher than prior to surgery (84.6% vs 3.7%, *p*= 0.000; and 78.2% vs 69.5%, *p*= 0.01, respectively), early miscarriage rate and preterm delivery rate were significantly lower (8.8% vs 80.6%, *p*= 0.000; and 7.0% vs 66.7%, *p*=0.000, respectively). After adjusting for body mass index, miscarriage history, and complications, multivariable logistic regression analysis revealed age ≥ 35 years and primary infertility as independent factors that affected postoperative clinical pregnancy (OR 4.025, 95% CI 2.063–7.851, *p*= 0.000; and OR 3.603, 95% CI 1.903–6.820, *p*= 0.000; respectively) and ongoing pregnancy (OR 3.420, 95% CI 1.812–6.455, *p*= 0.000; and OR 2.586, 95% CI 1.419–4.712, *p*= 0.002; respectively).

**Conclusions:**

Hysteroscopic metroplasty could lead to improved reproductive outcomes of women with septate uterus. Both age and primary infertility were independent factors for postoperative reproductive outcomes.

**Trial registration:**

Chi ECRCT20210343

## Introduction

A septate uterus is the most common congenital uterine anomaly, characterized by a septum that divides the uterus into two cavities (1). It is thought to be associated with poor reproductive outcomes including reduced live birth (37.9% vs 84.8%) and increased miscarriage (36.2–77.1% vs 9.1–16.7%) (2, 3). In one prospective study, the risk of miscarriage of women with septate uterus increased with each loss from approximately 11% to 40% after three or more pregnancy losses (4). A meta-analysis suggested that prevalence of septate uterus is 18% after two miscarriages and 17% after three (5). In addition, there is possible involvement of septate uterus in infertility of women. Studies suggested that approximately 3.5–8% (6, 7) of subfertile women, and 15.4–24.5% (6) of those with a history of miscarriage and infertility, had a septate uterus; in comparison, this prevalence is 2–3% in the general female population (8). The effective uterine volume reduction of the septate uterus results in miscarriage. However, as most miscarriages occur in the first trimester of pregnancy in the women with septate uterus (9), there may be other mechanisms such as abnormal function of the endometrium including endometrial receptivity and decidual transformation. Hysteroscopic metroplasty or hysteroscopic transcervical division of the uterine septum has been the standard treatment strategy to improve reproductive outcomes (10). Following surgery treatment, the miscarriage rate decreased from 94.3% to 10.4%, and live birth rate increased from 2.4% to 81.3% (2). Recently, some studies dispute this. A large cohort study of 257 patients and an international, multicenter randomized controlled trial of 68 patients showed that surgery treatment did not improve chance of conception (11, 12). Therefore, there are still controversies about the appropriateness of metroplasty (13). In addition, patients’ age, BMI, infertility and miscarriage history, and combinations may be the related demographic factors of fertility outcomes after surgery. A septate uterus does not always exist alone, and there are other possible uterine cavity disorders such as polyps, uterine adhesions, and endometriosis. However, there have been few studies of the factors related to reproductive outcomes of women after surgery.

This study aimed to 1) provide a general overview of reproductive outcomes after hysteroscopic metroplasty, 2) compare reproductive outcomes among women with a history of primary infertility and those without, and 3) analyze the demographic factors including patients’ age, BMI, infertility and miscarriage history, and combinations such as endometrial polyps, uterine adhesions, endometriosis and adenomyosis associated with reproductive outcomes of septate uterus after surgery.

## Methods

### Study population

Women with a septate uterus receiving hysteroscopic metroplasty in the Department of Minimally Invasive Gynecology at Beijing Obstetrics and Gynecology Hospital of Capital Medical University from February 2015 to February 2020 were retrospectively investigated and screened for inclusion in this study.

The inclusion criteria for women follow: (1) with septate uterus; (2) age 20–40 years; and (3) desiring pregnancy. The exclusion criteria for women follow: (1) with secondary surgery of residual uterine septum; (2) with submucosal uterine fibroids; and (3) with endometrial lesion (including endometrial intraepithelial neoplasia and endometrial tuberculosis).

Women with septate uterus were diagnosed by hysteroscopy or imaging examination. We conducted hysteroscopic metroplasty in patients with infertility, prior miscarriage, or adverse obstetrical outcome. In addition, because it was reasonable to consider surgical treatment following counseling regarding potential benefits, we also performed surgery on these women without previous poor reproductive outcomes. All patients signed informed consent for using their clinical data for this research.

This retrospective follow-up study was conducted according to the Declaration of Helsinki for Medical Research involving Human Subjects and was approved by the China Ethics Committee of Registering Clinical Trials (Chi ECRCT20210343).

### Data collection

We identified women retrospectively by searching electronic patient files, and clinical data were collected and examined. The following information was extracted by trained researchers: demographic, medical and pregnancy history including clinical pregnancy, miscarriage, live birth, and other uterine findings including intrauterine adhesions, endometrial polyps, endometriosis, and adenomyosis. Meanwhile, the patients’ further medical details and reproductive outcomes were obtained by telephone follow-up, and the follow-up timepoints were two years after hysteroscopic surgery.

### Operative procedures

The procedure is preferably performed in the early follicular phase of the menstrual cycle. Hysteroscopic metroplasty was conducted under laparoscopic guidance on all patients. Before the operation, the type of septum was again confirmed. Then the septum was cut along the midline starting from the outer part to the base of the septum. In addition, patients with complications including intrauterine adhesions, endometrial polyps, or endometriosis underwent hysteroscopy with resection of polyps, adhesiolysis, and laparoscopic excision of endometriosis. We performed second-look hysteroscopy at 3 months after metroplasty to evaluate the intrauterine cavity.

### Outcome measures and definitions

The primary outcome of the current study was live birth, defined as at least one live birth beyond 24 weeks of gestation. Secondary outcomes were ongoing pregnancy, clinical pregnancy, miscarriage, and preterm birth. Ongoing pregnancy was indicated by a viable intrauterine pregnancy of at least 12 weeks duration confirmed on an ultrasound scan (14). Clinical pregnancy referred to the presence of a gestational sac on ultrasound or by documentation of a birth, spontaneous abortion, or therapeutic abortion in the absence of ultrasound. Miscarriage was defined as intrauterine pregnancy loss before 24 weeks of gestation and early miscarriage was before 14 weeks of gestation. Preterm birth was defined as delivery of 24-37 weeks of gestation (15).

### Statistical analyses

Data analysis was performed using SPSS software, version 23.0. Independent proportions were compared using chi-squared test or Fisher’s exact probability method (when n ≤ 40 or T ≤ 1), whereas paired proportions were compared using McNemar chi-squared test. To evaluate factors of age, BMI, the type of septum, miscarriage history, infertility (primary and secondary), and complications (intrauterine adhesions, endometrial polyps, endometriosis, and adenomyosis) associated with reproductive outcomes after surgery treatment, we used a chi-square test for univariate analyses and multivariate logistic regression for multivariate analyses. Odds ratios (OR) with corresponding 95% confidence interval (95% CI) were calculated by adjusting for possible confounders.

## Results

### Patient characteristics

**Figure 1:**
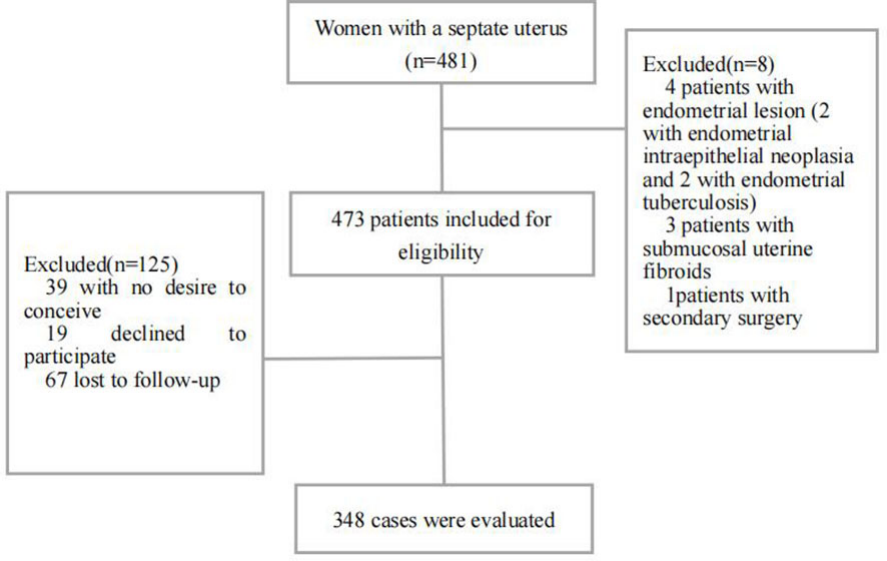
Flow chart of the screening of study population.

During the study period, 481 cases were searched; of these, eight cases were excluded and 125 were not followed. There were 348 cases ultimately included and completely followed (**Figure 1**). The median follow-up time after hysteroscopic metroplasty was 48 months (range 8–79 months). Mean age of included women was 30.1 ± 4.2 years and median age was 30 years (20–40 years). Mean BMI was 22.9 ± 3.6 kg/m^2^. There were 257 women (73.9%, 257/348) with partial septate uterus, while 91 (26.1%, 91/348) had complete septate uterus. There were 95 cases (27.3%, 95/348) combined with infertility (57 cases of primary infertility, 16.4%; and 38 cases of secondary infertility, 10.9%). There were 195 cases of spontaneous abortion (56.0%, 195/348); 124 cases (35.6%, 124/348) had one miscarriage and 71 cases (20.4%, 71/348) had two or more miscarriages;189 cases (54.3%, 189/348) had combinations: 107 cases (30.7%, 107/348) were combined with intrauterine adhesions, and 53 cases (15.2%, 53/348) were combined with endometrial polyps. Endometriosis was combined in 28 cases (8.0%, 28/348) and adenomyosis in 5 cases (1.4%, 5/348). Characteristics of women are shown in **Table 1**.

**Table 1:** Baseline characteristics of the participants.

Characteristics	n (%)
Age (Mean, 30.1 ± 4.2; median, 30)	
<35	300 (86.2%)
≥35	48 (13.8%)
BMI (Mean, 22.9 ± 3.6)	
<30	271 (77.9%)
≥30	77 (22.1%)
classification of septum	
partial septate uterus	257 (73.9%)
complete septate uterus	91 (26.1%)
combined Infertility	95 (27.3%)
Primary	57 (16.4%)
Secondary	38 (10.9%)
Previous miscarriage	195 (56.0%)
one	124 (35.6%)
two or more	71 (20.4%)
combined intrauterine adhesion	107 (30.7%)
combined endometrial polyps	53 (15.2%)
combined endometriosis	28 (8.0%)
combined adenomyosis	5 (1.4%)
mode of conception	
natural pregnancy	308 (88.5%)
artificial assisted reproduction	40 (11.5%)
without primary infertility and miscarriage history	96 (27.6%)

### Reproductive outcomes

Following surgery, the live birth rate was significantly higher than prior to surgery (84.6% vs 3.7%, *p* = 0.000), clinical pregnancy rate was significantly higher (78.2% vs 69.5%, *p* = 0.010), early miscarriage rate was significantly lower (8.8% vs 80.6%, *p* = 0.000), and preterm birth rate was significantly lower (7.0% vs 66.7%, *p*=0.000, **Table 2**). There was no statistical difference regarding live birth rate. In addition, of the 57 women with combined primary infertility, 28 (84.8%, 28/33) had live birth, 33 (57.9%, 33/57) achieved clinical pregnancy, 31 (54.4%, 31/57) had ongoing pregnancy, 3 (9.1%, 3/33) had early miscarriages, and 0 had preterm birth. The live birth rate between the two groups had no statistical significance. There was significant difference of clinical pregnancy and ongoing pregnancy between women with primary infertility and without (57.9% vs 82.1%, *p*=0.000; 54.4% vs 75.3%, *p*=0.001; respectively, **Table 3**).

**Table 2:** Comparison of reproductive outcomes between before and post hysteroscopic metroplasty.

	live birth			clinical pregnancy			early miscarriage			preterm birth		
	before operation	postoperation	P	before operation	postoperation	P	before operation	postoperation	P	before operation	postoperation	P
All participants	9 (3.7%)	230 (84.6%)	0.000	242 (69.5%)	272 (78.2%)	0.010	195 (80.6%)	24 (8.8%)	0.000	6 (66.7%)	16 (7.0%)	0.000
Complications excluded	5 (5.4%)	105 (86.8%)	0.000	92 (57.9%)	121 (76.1%)	0.001	73 (79.3%)	6 (5.0%)	0.000	3 (60.0%)	5 (4.8%)	0.000

**Table 3:** Comparison of postoperative fertility outcomes of patients with and without primary infertility.

Outcomes	with primary infertility n=57	without primary infertility n=291	P	Complications excluded n=159		
				with primary infertility n=36	without primary infertility n=123	P
live birth, n (% of clinical pregnancy)	28 (84.8%)	202 (84.5%)	1.000	16 (88.9%)	91 (88.3%)	1.000
clinical pregnancy, n (%)	33 (57.9%)	239 (82.1%)	0.000	18 (50.0%)	103 (83.7%)	0.000
ongoing pregnancy, n (%)	31 (54.4%)	219 (75.3%)	0.001	18 (50.0%)	93 (75.6%)	0.003
early miscarriage, n (% of clinical pregnancy)	3 (9.1%)	21 (8.8%)	1.000	1 (5.6%)	5 (4.9%)	1.000
preterm birth, n (% of live birth)	0 (0%)	16 (7.9%)	0.230	0 (0%)	5 (5.5%)	1.000

When the complications (intrauterine adhesions, endometrial polyps, endometriosis, and adenomyosis) had been excluded, the live birth rate was significantly higher than prior to surgery (86.8% vs 5.4%, *p*=0.000), clinical pregnancy rate was significantly higher (76.1% vs 57.9%, *p*=0.001), early miscarriage rate was significantly lower (5.0%vs. 79.3%, *p*=0.000), and preterm birth rate was significantly lower (4.8% vs 60.0%, *p*=0.000) in the 159 women (**Table 2**). Of the 36 women with primary infertility, 16 (88.9%, 16/18) had live birth, 18 (50.0%,18/36) had clinical pregnancy, 18 (50.0%, 18/36) had ongoing pregnancy, 1 (5.6%, 1/18) had early miscarriage, and 0 had preterm live birth. No significant difference was found for the rate of live birth rate. There was significant difference of clinical pregnancy and ongoing pregnancy between women with primary infertility and without (50.0% vs 83.7%, *p*=0.000; 50.0% vs 75.6%, *p*=0.003; respectively). The reproductive outcomes are summarized in **Table 3**.

### Factors associated with reproductive outcomes after surgery

The factors of age, BMI, the type of septum, miscarriage history, infertility (primary and secondary), and complications (intrauterine adhesions, endometrial polyps, endometriosis, and adenomyosis) were analyzed. Univariate analysis showed that age, combined primary infertility, and prior miscarriage history may be associated with clinical pregnancy after surgery. All these factors had no significant effect on live birth. The details are given in **Table 4**.

**Table 4:** Univariate analysis of reproductive outcomes after hysteroscopic metroplasty.

Variable	Live birth		Clinical pregnancy		Early miscarriage		Ongoing pregnancy		Preterm delivery	
	χ^2^	P value	χ^2^	P value	χ^2^	P value	χ^2^	P value	χ^2^	P value
Age	——*	1.000	22.182	0.000	——*	0.471	——*	1.000	——*	1.000
BMI	0.315	0.574	1.710	0.191	0.679	0.410	0.000	0.987	0.256	0.613
Type of septum	0.248	0.618	6.864	0.009	0.247	0.619	0.557	0.455	1.683	0.194
Infertility	0.002	0.964	19.734	0.000	0.011	0.915	0.000	1.000	1.518	0.218
Primary	——*	1.000	16.401	0.000	0.000	1.000	——*	1.000	——*	0.232
secondary	——*	1.000	2.371	0.124	——*	1.000	——*	0.452	——*	1.000
miscarriage	0.158	0.924	6.279	0.043	1.008	0.604	0.923	0.630	2.962	0.227
combined intrauterine adhesion	2.325	0.127	0.032	0.859	1.544	0.214	0.386	0.534	0.004	0.948
combined endometrial polyps	0.390	0.533	0.024	0.878	0.000	1.000	0.000	1.000	0.431	0.511
combined endometriosis	——*	0.560	——*	0.159	——*	0.254	——*	0.130	——*	0.648
combined adenomyosis	——*	0.491	——*	1.000	——*	0.310	——*	1.000	——*	1.000
mode of conception	——*	0.596	0.012	0.914	——*	0.496	——*	0.293	——*	0.406

*Fisher’s exact probability method.

**Figure 2:**
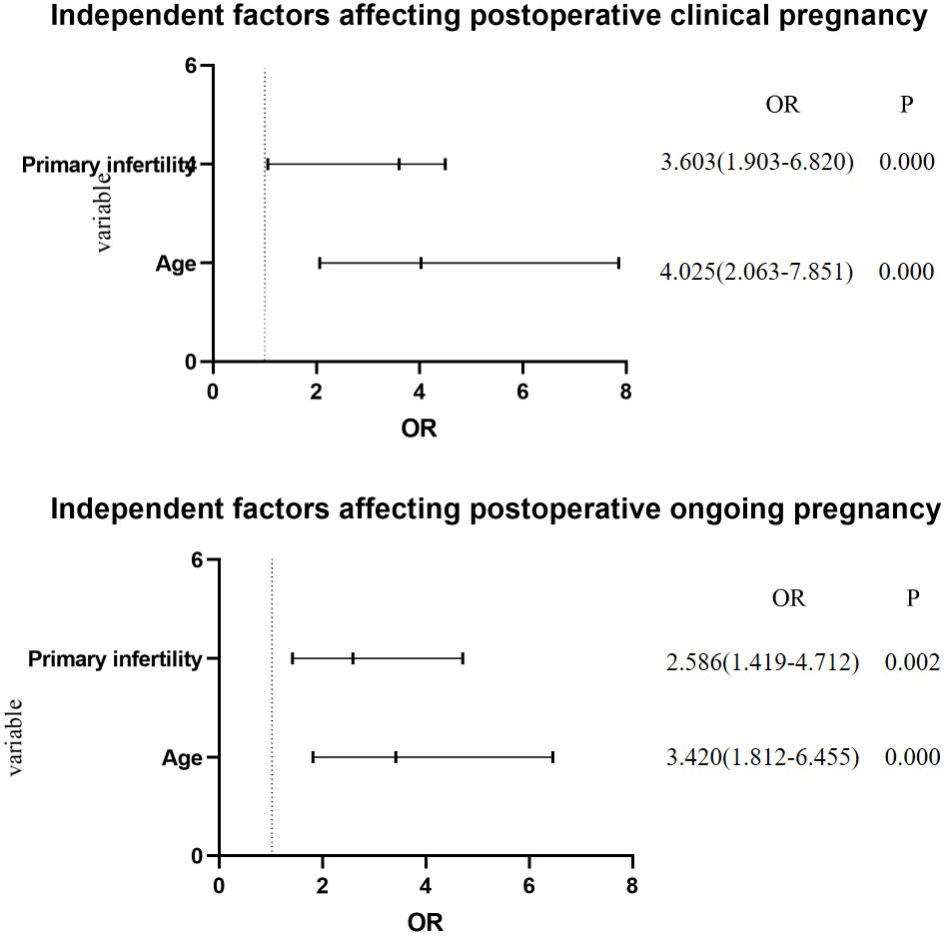
Independent factors.

We further analyzed independent risk factors using a multivariate logistic regression analysis model; after adjusting for prior miscarriage history and complications, we found age ≥ 35 years and primary infertility had significant associations with lower clinical pregnancy (OR 4.025, 95% CI 2.063–7.851, *p* = 0.000; and OR 3.603, 95% CI 1.903–6.820, *p* = 0.000; respectively) and lower ongoing pregnancy (OR 3.420, 95% CI 1.812–6.455, *p* = 0.000; and OR 2.586, 95% CI 1.419–4.712, *p* = 0.002; respectively, **Figure 2**, **Table 5**).

**Table 5:** Multivariate analysis of reproductive outcomes after hysteroscopic metroplasty.

Variable	postoperative clinical pregnancy			postoperative ongoing pregnancy		
	n	OR (95%CI)	P value	n	OR (95%CI)	P value
age		4.025 (2.063−7.851)	0.000		3.603 (1.903−6.820)	0.000
<35	247			227		
≥35	25			23		
Primary infertility		3.603 (1.903-6.820)	0.000		2.586 (1.419-4.712)	0.002
without	239			219		
with	33			31		

## Discussion

Our main findings are as follows: (1) live birth, clinical pregnancy, and early miscarriage rate were significantly improved following surgery treatment in patients with septate uterus with or without complications; (2) patients with primary infertility history had a lower clinical pregnancy rate compared to those who were not; and (3) of the possible factors associated with reproductive outcomes, age ≥ 35 years and primary infertility history may be independent risk factors for postoperative clinical pregnancy. Our study finally included 348 women, which was the largest sample size to date on this topic. Following surgery, live birth rate increased from 3.7% to 84.6%, clinical pregnancy rate increased from 69.5% to 78.2%, and early miscarriage decreased from 80.6% to 8.8%. In the previous studies, the reported live birth rate range was 76.2–81.3% after surgery compared to 2.4–4.3% prior to surgery (16). These other studies support that surgical treatment has a positive effect on the reproductive prognosis.

However, there is still controversy in regard to this topic. Some studies (11–13, 17) found no significant difference in reproductive outcomes between the two groups. A Cochrane review published in 2011 reported that there was no evidence for hysteroscopic metroplasty in women with recurrent miscarriage and a septate uterus, and advised against offering this intervention as routine practice. Recently, an international multicentre cohort study (11)was performed and included a total of 257 women with septate uterus: 151 underwent septum resection and 106 had expectant management. They suggested that surgical treatment did not improve reproductive outcomes. It is important that demographic factors including age, BMI, reproductive history, and comorbidity may be risk factors affecting the subsequent reproductive outcomes in women with septate uterus after surgical treatment. In our study, we performed univariate and multivariate analyses to find potential influencing factors. This is the first study to analyze risk factors associated with postoperative reproductive outcomes in women with septate uterus who desire to conceive. The results suggest that both age and primary infertility history were independent risk factors for postoperative reproductive outcome.

The surgery indication of septate uterus is not uniform, and current guidelines have different recommendations. The ASRM and NICE guidelines (18) recommend removing the intrauterine septum, whereas the ESHRE (19) and RCOG guidelines (20) recommend not performing surgery and suggest that the procedure should be evaluated in future studies. Some scholars suggest that women should not undergo extensive evaluation after a single first trimester or early second trimester miscarriage, given that these are relatively common with only a modestly increased risk of recurrence (21–23). The individualized treatment for patients of different ages and with comorbidities was ignored. Our findings provide some evidence for the clinical practice. For example, the indication may be relaxed for older women, and women with combined intrauterine adhesions, endometrial polyps, endometriosis, or adenomyosis. There may be potential benefits to consider septum resection after counseling regarding potential risks of the procedure in women. In addition, second- generation techniques have been used in hysteroscopy with good results. These new technological advances in the treatment of septate uterus have demonstrated sufficient efficacy in terms of reproductive outcomes (24–26).

However, the association between septate uterus and infertility is still unclear. Studies that evaluated women with primary infertility or unexplained infertility (2) showed an improved pregnancy rate followed surgery. In our study, we found the postoperative reproductive outcomes of women with primary infertility were improved but their clinical pregnancy was lower than women without it. We hypothesize that surgery could restore normal anatomy but not completely restore normal function. Initially, a uterine septum was believed to be predominantly fibrous tissue. However, magnetic resonance imaging and biopsy specimens suggest that the septum is primarily composed of muscle fibers and less connective tissue. One recent systematic literature review suggested that there was a lower number of glandular and ciliated cells and lower expression of HOXA10 genes and VEGF receptor genes in the endometrial lining of the septum, which possibly account for the poor reproductive outcomes (27). The vascularization, myometrium, and endometrium of septum are similar to the normal uterine wall. Further study should be performed to research the infertility mechanism and more effective treatment of septate uterus.

Our study has some limitations. The retrospective trial was at high risk of bias due to the before/after design. Although the factors of age, BMI, the type of septum, reproductive history, and comorbidities were analyzed to determine the influencing variables, there were other confounding factors such as women who were lost to follow-up, selection bias, and some unidentified factors. In addition, we found that early pregnancy accounted for 83.3% of all miscarriage in patients with septate uterus, so so we took early miscarriage as the outcome indicator of the study.

## Conclusions

Hysteroscopic metroplasty could lead to improved reproductive outcomes of women with septate uterus, including those with miscarriage history and combined intrauterine adhesions, endometrial polyps, endometriosis, or adenomyosis. Both age and primary infertility were independent risk factors for postoperative reproductive outcomes. These provide some basis for the establishment of surgery treatment indication and personalized treatment strategy for women with septate uterus who desire to conceive.

## Data availability statement

The raw data supporting the conclusions of this article will be made available by the authors, without undue reservation.

## Ethics statement

The studies involving human participants were reviewed and approved by the China Ethics Committee of Registering Clinical Trials. The patients/participants provided their written informed consent to participate in this study.

## Author contributions

YC, MS, and HD conceived and designed the study. YC, MS, SW, and ZG searched and collected data. YC, MS, and ZG analyzed data. YC, MS, SW wrote the manuscript. All authors contributed to the article and approved the submitted version.
